# Association of remnant cholesterol with depression among US adults

**DOI:** 10.1186/s12888-023-04770-4

**Published:** 2023-04-17

**Authors:** Yang Wang, Ruhua Shen

**Affiliations:** Department of Cardiovascular Surgery, Division of Life Sciences and Medicine, The First Affiliated Hospital of USTC, University of Science and Technology of China, Hefei, Anhui 230001 China

**Keywords:** Remnant cholesterol, Depression, NHANES

## Abstract

**Background:**

Remnant cholesterol is receiving increasing attention because of its association with various diseases. However, there have been no studies on remnant cholesterol levels and depression.

**Methods:**

A cross-sectional analysis was performed based on the National Health and Nutrition Examination Survey (NHANES) 2005–2016. Depression was assessed using a Patient Health Questionnaire (PHQ-9). Fasting remnant cholesterol was calculated as the total cholesterol minus high-density lipoprotein cholesterol (HDL-C) minus low-density lipoprotein cholesterol (LDL-C). Logistic regression analysis with sampling weights was used to examine the association between remnant cholesterol concentration and depression.

**Results:**

Among 8,263 adults enrolled in this study (weighted mean age, 45.65 years), 5.88% (weighted percentage) had depression. Compared to the participants without depression, those with depression had higher concentration of remnant cholesterol (weighted mean, 26.13 vs. 23.05, P < 0.001). There was a significant positive relationship between remnant cholesterol concentration and depression and multivariable-adjusted OR with 95% CI was 1.49 (1.02–2.17). Among the subgroup analyses, remnant cholesterol concentration was positively associated with depression among participants less than 60 years (OR, 1.62; 95% CI, 1.09–2.42), male (OR, 2.02; 95% CI, 1.01–4.05), BMI under 30 (OR, 1.83; 95% CI, 1.14–2.96), and those with diabetes (OR, 3.88; 95% CI, 1.43–10.49).

**Conclusions:**

Remnant cholesterol concentration positively correlated with depression, suggesting that a focus on remnant cholesterol may be useful in the study of depression.

## Introduction

Depression is a global disease that imposes a huge burden on both individuals and the society. The World Health Organization (WHO) reports that approximately 4.4% of the world’s population suffers from depression [1]. The number of reported cases of depression increased globally by 49.86% from 1990 to 2017[2]. depression is associated with an increased risk of several diseases, including coronary heart disease (CHD)[3], hypertension [4], diabetes [5], obesity [6], and stroke [7]. There are multiple risk factors for depression such as stress [8], behavioral patterns [9], and socio-demographic factors [10, 11]. Additionally, some studies have found that cholesterol may be associated with depression [12, 13].

Remnant cholesterol (RC), also known as triglyceride-rich cholesterol, consists mainly of very-low-density lipoproteins (VLDLs), intermediate-density lipoproteins (IDLs), and chylomicron remnants in fasting or non-fasting state [14]. RCis gaining attention because of its close association with cardiovascular diseases [15]. Epidemiological studies have found that RC is also associated with hypertension [16], diabetes mellitus and its complications [17, 18], nonalcoholic fatty liver disease [19], and chronic kidney disease [20].

Several studies have shown that chronic low-grade inflammation contributes to the pathophysiological changes associated with major depression [21, 22]. A bidirectional association between inflammation and depression has been reported in several studies on adolescents with major depression [22]. Currently, depression is treated using either non-pharmacological treatments, pharmacological treatments, or a combination of both [23]. In the treatment of depression, it has been found that a significant proportion of patients do not respond adequately to the application of various treatments, or show any response at all [24]. Further studies have confirmed the association between inflammation and non-responsiveness to antidepressant treatment [25]. It is evident that inflammation is closely involved in the development of depression. High levels of RC in serum is more likely to permeate the arterial wall, be captured and absorbed by macrophages, leading to rapid formation of foam cells [26]. A causal relationship between RC and low-grade chronic inflammation has been found [27]. Based on these findings, RC may be involved in the development and progression of depression through chronic inflammation. However, there is little evidence of the relationship between RC level and depression from population-based epidemiological studies. This study aimed to investigate the potential relationship between RC and depression in the general population.

## Methods

**Fig. 1:**
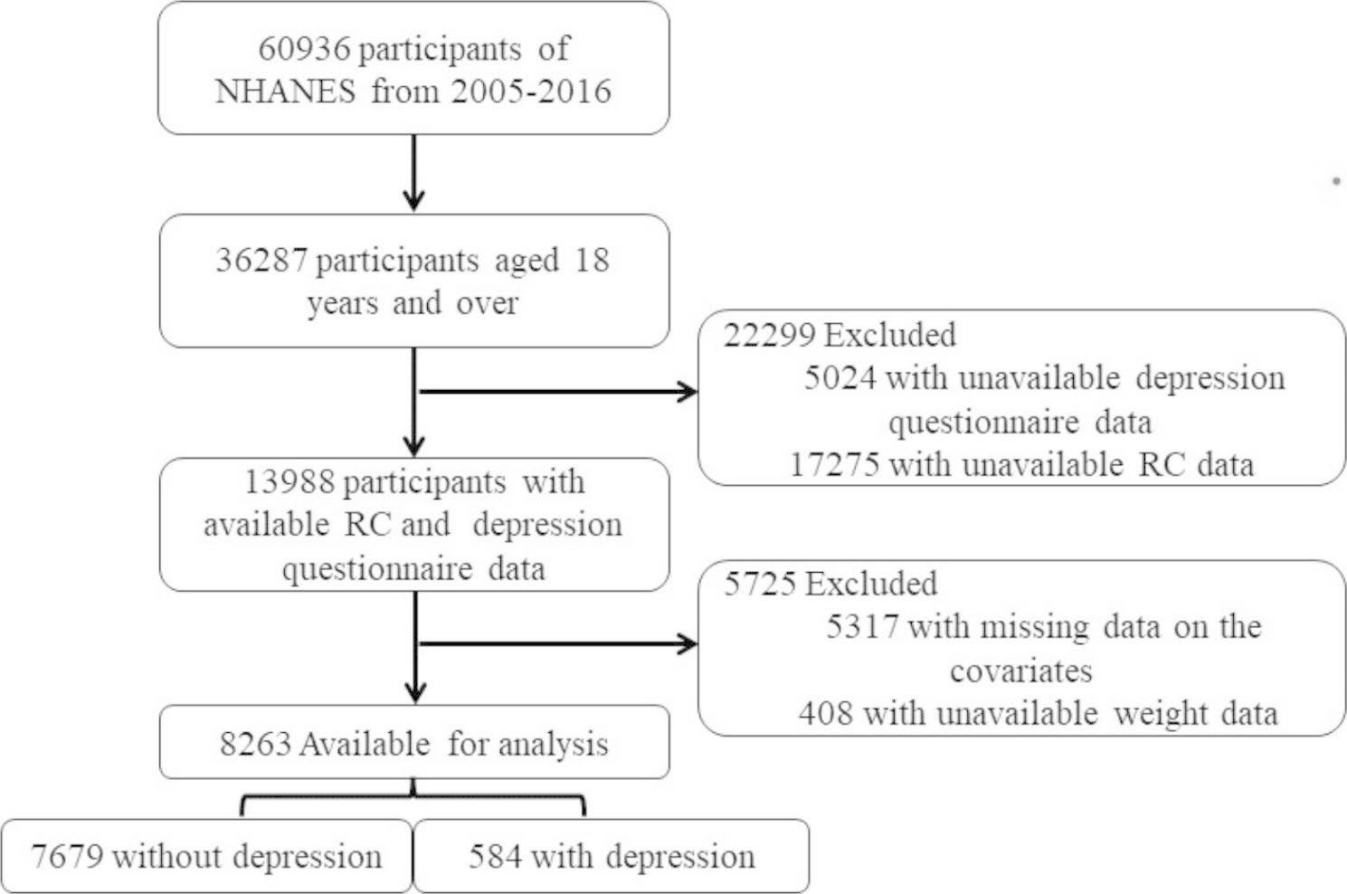
Flow chart of the screening process for the selection of eligible participants

NHANES is a nationally representative survey administered by the National Center for Health Statistics (NCHS) in which complex survey design and population-specific sample weights have been applied to evaluate health or nutritional status of non-institutionalized population in the United States. The assessment methodology consists of a range of sampled home interviews and standardized physical examination at a mobile examination center (MEC). We extracted and analyzed information gathered from adult participants during six 2-year NHANES cycles (2005–2016). Since this study used publicly available, non-identifying data published by the Centers for Disease Control and Prevention, Institutional Review Board approval was not required. The following exclusion criteria were used: participants who were younger than 18 years; missing data on the diagnosis of depression or calculation of RC or covariates; weight data not available (fasting weights not available or value of 0). A flowchart of this process is presented in Fig. 1.

### Depression

Depression was assessed using the Patient Health Questionnaire (PHQ-9), a 9-item screening tool that contained questions about the frequency of depressive symptoms in the previous 2 weeks. Response categories for the nine-item instrument included “not at all,“ “several days,“ “more than half the days,“ and “nearly every day” and were given scores from 0 to 3. The instrument incorporates the DSM-IV depression diagnostic criteria [28]. The total PHQ-9 score ranged from 0 to 27. In this study, those with a total PHQ-9 score of ≥ 10 were considered to have major depression. Previous studies have shown that the sensitivity and specificity of this clinical cut-off for major depression is 88%[29, 30].

### Remnant cholesterol

RC was calculated from the patient’s standard lipid profile in the fasting state, which was the total cholesterol (TC) minus low-density lipoprotein cholesterol (LDL-C) minus high-density lipoprotein cholesterol (HDL-C)[31].

### Covariates

Potential covariates included in the study were age, sex, race/ethnicity, poverty income ratio (PIR), education level, marital status, HDL-C, LDL-C, estimated glomerular filtration rate (eGFR, ml/min/1.73 m^2^), body mass index (BMI, kg/m2), physical activity (min/week), smoking status, alcohol consumption, chronic obstructive pulmonary disease (COPD), cardiovascular disease (CVD), diabetes, and hypertension. Self-reported race/ethnicity was categorized into the following four races/ethnicities: non-Hispanic Black, non-Hispanic White, Mexican American, and Other race. Educational level was divided into three levels: high school or less, some college, and college graduate or higher. Marital status was sorted into four groups: married, never married, living with a partner, and others.

EGFR expression was calculated using the Chronic Kidney Disease Epidemiology Collaboration creatinine equation [32]. Physical activity was defined as the total number of minutes of activity per week. These activities included walking or cycling, tasks around the house or yard, muscle-strengthening activities, work activities, and recreational activities. Smoking status was categorized into the following three groups: never (smoked less than 100 cigarettes in life), former (smoked more than 100 cigarettes in life and does not smoke at all now), and current (smoked more than 100 cigarettes in life and smokes some days or every day). Alcohol consumption was assessed by using a question: “In any one year, have you had at least 12 drinks of any type of alcoholic beverage?” Participants who answered ‘yes’ were identified as alcohol drinkers. COPD was diagnosed by any one of the following criteria: have been told by a doctor or health professional that they have COPD or emphysema; FEV1/FVC < 0.7 post-bronchodilator; age 40 years or older; history of smoking or chronic bronchitis; and use of selective phosphodiesterase-4 inhibitors, mast cell stabilizers, leukotriene modulators, or inhaled corticosteroids. Participants who had been notified of any of the following conditions: congestive heart failure, coronary artery disease, angina, heart attack, and stroke were considered to have CVD. Participants having diabetes were identified by having any of the following conditions: have been told by a doctor or health professional that they have diabetes, glycohemoglobin ≥ 6.5%, fasting glucose ≥ 7.0 mmol/l, random blood glucose ≥ 11.1 mmol/l, two-hour OGTT blood glucose ≥ 11.1 mmol/l, and use of diabetes medication or insulin. Hypertension in participants was defined based on any of the following: ever been told by a doctor or a health professional that had hypertension, mean systolic blood pressure ≥ 130 mmHg, and mean diastolic blood pressure ≥ 80 mmHg.

### Statistical analyses

The complex sampling design and sampling weights of NHANES were incorporated in our analysis. The sampling weight was computed by the following formula: fasting subsample 12-year mobile examination center (MEC) weight = fasting subsample 2-year MEC weight/6. Continuous data are described as weighted mean (standard error), whereas categorical data are summarized as weighted percentages.

RC data was divided into quartiles, and the lowest quartile was used as the reference category. The association between RC and the risk of depression was analyzed using weighted logistic regression models, and 95% confidence intervals (CIs) and odds ratios (ORs) were calculated. No covariates were adjusted in the unadjusted model. Age and sex were adjusted for in Model 1. Model 2 was adjusted for race/ethnicity, marital status, PIR, education level, BMI, smoking status, alcohol consumption, eGFR, physical activity, HDL-C level, and LDL-C level based on Model 1. Model 3 was further adjusted for COPD, CVD, diabetes, and hypertension. Subgroup analyses were presented with logistic regression models based on age, sex, BMI, and diabetes. In addition, several sensitivity analyses were performed to specifically assess the robustness of our conclusion. First, we selected 24 mg/dL (0.62 mmol/L) as the cut-off point for RC, which was studied as a binary variable [17]. Second, we performed an analysis based on unweighted raw data by using inverse probability of treatment weighting (IPTW) to tackle potential confounders. Third, participants who used antidepressant medications in the baseline data were excluded. P < 0.05 was considered as statistically significant. All statistical analyses were performed using R software (version 4.1.3).

## Results

### Characteristics of the participants

The NHANES 2005–2016 sample included 36,287 adult participants aged ≥ 18 years. Among them 28,024 individuals were excluded because of missing or unavailable data concerning depression, RC, covariates, and weight, meanwhile the remaining 8,263 individuals were considered for the analysis. A total of 584 participants (5.88%, weighted percentage) had depression, and 7,679 (94.12%, weighted percentage) did not have depression. Table 1 presents the characteristics of the study population. Based on the weighted analysis, the mean age of the participants was 45.65 years and 48.34% were female. Participants with depression were more probably to be female (61.48 vs. 47.52%; p < 0.001), with higher RC (mean = 26.13 vs. 23.05, p < 0.001), BMI (mean = 30.14 vs. 28.39, p < 0.001), current smoking (41.26 vs. 18.91%, p < 0.001), and have a lower PIR (mean = 2.05 vs. 3.18, p < 0.001). Statistically significant differences were also found in the weighted percentages of participants with and without depression regarding race/ethnicity, education level, marital status, COPD, CVD, diabetes, and hypertension.

**Table 1:** Weighted baseline characteristics of patients with or without depression

Characteristic	total	Without Depression	With Depression	
		94.12%	5.88%	p value
Age (year)	45.65(0.31)	45.67(0.33)	45.34(0.83)	0.7
Sex (%)				< 0.001
Female	48.34	47.52	61.48	
Male	51.66	52.48	38.52	
Race/ethnicity (%)				0.002
Non-Hispanic black	10.03	9.75	14.48	
Non-Hispanic white	71.46	71.86	64.98	
Mexican American	7.17	7.13	7.79	
Other race	11.34	11.25	12.75	
PIR	3.12(0.04)	3.18(0.04)	2.05(0.11)	< 0.001
Education level (%)				< 0.001
High school or less	35.41	34.47	50.47	
Some college	31.98	31.76	35.58	
College graduate or higher	32.61	33.77	13.95	
Marital status (%)				< 0.001
Married	57.12	58.4	36.72	
Never married	18.81	18.56	22.84	
Living with partner	8.42	8.24	11.23	
Other	15.65	14.8	29.21	
HDL-C (mg/dl)	54.89(0.28)	54.98(0.29)	53.36(0.92)	0.09
LDL-C (mg/dl)	115.08(0.54)	115.02(0.58)	116.05(1.69)	0.58
RC (mg/dl)	23.23(0.23)	23.05(0.23)	26.13(0.81)	< 0.001
eGFR (ml/min/1.73m2)	96.31(0.39)	96.17(0.39)	98.69(1.20)	0.03
BMI (kg/m2)	28.49(0.11)	28.39(0.11)	30.14(0.32)	< 0.001
Physical activity (min/week)	1027.31(27.56)	1027.54(27.52)	1023.61(73.91)	0.96
Smoking status (%)				< 0.001
Never	54.51	55.58	37.44	
Former	25.26	25.51	21.3	
Current	20.22	18.91	41.26	
Alcohol consumption (%)				0.35
No	20.47	20.38	21.93	
Yes	79.53	79.62	78.07	
COPD (%)				< 0.001
no	95.46	95.69	91.65	
yes	4.54	4.31	8.35	
CVD (%)				< 0.001
no	92.66	93.09	85.91	
yes	7.34	6.91	14.09	
Diabetes (%)				< 0.001
no	87.61	88.03	80.98	
yes	12.39	11.97	19.02	
Hypertension (%)				< 0.001
no	65.71	66.26	56.94	
yes	34.29	33.74	43.06	

### Association of RC with depression

The association between RC and depression is presented in Table 2. Unadjusted Model with RC adjusted alone, showed a statistically significant positive relationship between RC and depression, the OR with 95% CI in the highest versus lowest quartets was 1.80(1.34–2.42). In Model 1, after adjusting for RC, age, and sex, the OR with 95% CI of depression in the highest versus lowest quartets was 2.07(1.54–2.79). When further adjusted for race/ethnicity, marital status, PIR, education level, BMI, smoking status, alcohol consumption, eGFR, physical activity, HDL-C, and LDL-C based on Model 1, the OR with 95% CI was 1.55(1.06–2.25) in Model 2. The association between RC and depression did not change substantially in Model 3 (fully adjusted model; OR, 1.49; 95% CI, 1.02–2.17).

**Table 2:** Association between remnant cholesterol and depression among US adults

	Unadjusted Model			Model 1			Model 2			Model 3	
RC	OR (95% CI)	p value		OR (95% CI)	p value		OR (95% CI)	p value		OR (95% CI)	p value
1st	1 (Referent)			1 (Referent)			1 (Referent)			1 (Referent)	
2nd	1.23(0.84,1.80)	0.28		1.30(0.89,1.89)	0.17		1.08(0.73,1.58)	0.7		1.06(0.72,1.57)	0.76
3rd	1.16(0.82,1.64)	0.41		1.28(0.91,1.81)	0.16		1.05(0.72,1.53)	0.82		1.02(0.69,1.50)	0.93
4th	1.80(1.34,2.42)	< 0.001		2.07(1.54,2.79)	< 0.001		1.55(1.06,2.25)	0.02		1.49(1.02,2.17)	0.04
p for trend	< 0.001			< 0.001			0.03			0.04	

Model 1: adjusted for age and sex;

Model 2: adjusted for age, sex, race/ethnicity, marital status, PIR, education level, BMI, smoking status, alcohol consumption, eGFR, physical activity, HDL-C, LDL-C;

Model 3: further adjusted for COPD, CVD, diabetes and hypertension based on Model 2

### Subgroup analyses

The results of the subgroup analyses are showed in Table 3. RC was significantly, positively, associated with depression among participants aged < 60 years (OR, 1.62; 95% CI, 1.09–2.42), male (OR, 2.02; 95% CI, 1.01–4.05), BMI under 30 (OR, 1.83; 95% CI, 1.14–2.96), and those with diabetes (OR, 3.88; 95% CI, 1.43–10.49) exposed to the highest quartets. There was no association with females, participants aged > 60 years, or those without diabetes or in the obesity subgroups.

**Table 3:** Subgroup analyses stratifed by sex, age, BMI and diabetes

	RC	1st		2nd			3rd			4th			p for trend	p for interaction
				OR (95% CI)	p value		OR (95% CI)	p value		OR (95% CI)	p value			
Sex														0.75
Female		1 (Referent)		1.04(0.67,1.61)	0.86		1.15(0.74,1.79)	0.54		1.19(0.72,1.96)	0.49		0.4	
Male		1 (Referent)		1.10(0.58,2.12)	0.76		0.85(0.42,1.72)	0.64		2.02(1.01,4.05)	0.05		0.04	
Age														0.94
< 60		1 (Referent)		1.21(0.80,1.83)	0.37		1.16(0.74,1.82)	0.51		1.62(1.09,2.42)	0.02		0.03	
≥ 60		1 (Referent)		0.54(0.22,1.33)	0.18		0.53(0.21,1.33)	0.17		0.90(0.32,2.57)	0.84		0.81	
BMI														0.23
< 30		1 (Referent)		1.02(0.65,1.59)	0.94		0.91(0.54,1.53)	0.71		1.83(1.14,2.96)	0.01		0.03	
≥ 30		1 (Referent)		1.25(0.70,2.24)	0.45		1.11(0.59,2.11)	0.74		1.16(0.63,2.15)	0.63		0.83	
diabetes														0.06
no		1 (Referent)		1.02(0.66,1.57)	0.94		0.94(0.62,1.41)	0.75		1.36(0.87,2.14)	0.17		0.2	
yes		1 (Referent)		2.63(0.78, 8.85)	0.494		2.70(1.04, 6.98)	0.11		3.88(1.43,10.49)	0.01		0.01	

adjusted for age, sex, race/ethnicity, marital status, PIR, education level, BMI, smoking status, alcohol consumption, eGFR, physical activity, HDL-C, LDL-C, COPD, CVD, diabetes and hypertension except the stratification factor itself

### Sensitivity analyses

Table 4 presents the results of the sensitivity analysis. After selection of 24 mg/dL (0.62 mmol/L) as the cut-off point for the RC, RC was associated with depression (OR, 1.30; 95% CI, 1.01–1.68). After IPTW (unweighted n = 5028), the OR with 95% CI in the highest versus lowest quartets was 1.38 (1.07–1.79). After excluding participants who used antidepressant medication, the results were not significant; however, the direction was consistent with previous results when the highest quartile was compared with the lowest quartile (*p* for trend = 0.04).

**Table 4:** Sensitivity analyses

	OR (95% CI)	p value		p for trend
RC				
< 24	1 (Referent)			
≥ 24	1.30(1.01,1.68)	0.04		
Inverse probability treatment weighted analyses				
1st	1 (Referent)			
2nd	1.24(0.94,1.63)	0.13		
3rd	1.11(0.85,1.47)	0.45		
4th	1.38(1.07,1.79)	0.02		< 0.001
Excluding participants take antidepressants				
1st	1 (Referent)			
2nd	1.08(0.70,1.67)	0.73		
3rd	1.11(0.71,1.74)	0.63		
4th	1.60(0.99,2.59)	0.05		0.04

adjusted for age, sex, race/ethnicity, marital status, PIR, education level, BMI, smoking status, alcohol consumption, eGFR, physical activity, HDL-C, LDL-C, COPD, CVD, diabetes and hypertension

## Discussion

As far as we know, this is the first to investigate the relationship between RC and the risk of depression in a nationally representative population of U.S. samples. We found a positive association between RC concentration and the incidence of depression among American adults. Subgroup analysis showed that the OR of the association between RC and depression was higher among males, participants younger than 60 years, non-obese, and those with diabetes. All the sensitivity analyses provided further support for the robustness of our results.

Cholesterol is an important component of mammalian cell membranes and is a precursor for the synthesis of bile acids and steroid hormones [33]. However, excess cholesterol exhibits ubiquitous toxicity owing to its ability to cause cellular dysfunction and death [34]. Studies have shown that excessive cholesterol accumulation plays a key role in the pathogenesis of a variety of diseases including liver disease [35], diabetes [36], chronic kidney disease [37], cognitive impairment [38], osteoporosis [39], osteoarthritis [40], pituitary-thyroid axis dysfunction [41], immune disorders [42], and COVID-19[43]. Several studies have investigated the association between cholesterol and depression. However, studies focusing on total cholesterol, low-density cholesterol, and high-density cholesterol have produced inconsistent and sometimes conflicting results [44–48]. RC, an important component of cholesterol, accounts for one-third of total cholesterol [49] and is the source of 50% of cholesterol in atherosclerotic plaques [50]. Increasing number of studies have found an association between RC and a variety of diseases [15–20]. Our study confirms that elevated RC is positively associated with the risk of depression.

The exact mechanism by which RC is associated with depression requires further investigation. The following potential pathways maybe involved: First, elevated RC may be involved in the pathogenesis of depression through the activation of hypothalamic-pituitary-adrenal (HPA) axis. High serum concentration of RC contributes to increased penetration into the arterial wall, wherein RC is more readily captured and absorbed by macrophages than LDL, which in turn leads to faster foam cell formation [26]. Macrophage foam cells express interleukin 6 (IL-6) and circulating IL-6 stimulates the HPA axis [51]. HPA axis changes are associated with depression and impaired cognitive function [52–54]. Second, RC could be linked to depression through the production of cytokines that act on the nerve cells. Peripheral cytokines can affect neurons and supporting cells directly after crossing the blood-brain barrier or through afferent pathways [55]. One study has shown that interferon gamma and interleukin 2 can trigger depression when used therapeutically [56]. Increased levels of IL-6 in childhood increase the risk of depression later in life, further supporting the role of inflammation in the pathogenesis and exacerbation of depression [57]. Third, RC-induced low-grade inflammation and endothelial dysfunction may lead to cerebral microvascular dysfunction. Studies have shown that RC can cause hypo-inflammation [27] and endothelial diastolic dysfunction [58], which is confirmed to be one of the common mechanisms of arterial stiffness [59]. Large artery stiffness may lead to cerebral microvascular dysfunction [60]. Population-based data suggests that greater stiffness of the carotid arteries is associated with a higher risk of depressive symptoms [61].

### Limitations

This study has several limitations. First, this was a cross-sectional study, which allowed us to describe the existence of this condition but we could not derive causality. Therefore, there is a need for prospective studies in the future. Second, the cholesterol data used in this study was fasting data while non-fasting data has not been studied. However, systematic studies comparing fasting and non-fasting samples have shown small differences in most lipid parameters [62–64]. For general risk screening, non-fasting lipid samples appear to have the same prognostic value as fasting samples [65]. Third, since there is no uniform clinical method to measure RC, RC calculated indirectly from TC minus LDL-C minus HDL-C is different from RC measured directly using nuclear magnetic resonance [66]. Nevertheless, this indirect approach via calculation does not incur additional costs and is expected to be widely used in clinical practice to facilitate clinical research. Fourth, the associations we investigated may have been influenced by multiple confounding factors. Although we adjusted for relevant covariates as much as possible, there are probably more relevant covariates that we did not adjust for.

## Conclusion

This cross-sectional study showed a positive association between fasting RC concentration and depression in adults in the United States. Future prospective studies are needed to confirm our findings and investigate the efficacy of RC-lowering therapy in patients with depression.

## Data Availability

The National Health and Nutrition Examination Survey dataset is publicly available at the National Center for Health Statistics of the Center for Disease Control and Prevention (https://www.cdc.gov/nchs/nhanes/index.htm).

## Abbreviations

RC: Remnant cholesterol
PIR: Poverty income ratio
HDL-C: High-density lipoprotein cholesterol
LDL-C: Low-density lipoprotein cholesterol
BMI: Body mass index
eGFR: Estimated glomerular filtration rate
COPD: Chronic obstructive pulmonary disease
CVD: Cardiovascular disease
CI: Confidence interval
OR: Odds ratio
NHANES: National health and nutrition examination surveys
NCHS: National Center for Health Statistics
PHQ-9: Patient Health Questionnaire
WHO: World Health Organization
CHD: Coronary heart disease
VLDLs: Very-low-density lipoproteins
IDLs: Intermediate-density lipoproteins
MEC: Mobile examination center
TC: Total cholesterol
IPTW: Inverse probability of treatment weighting
HPA axis: Hypothalamic-pituitary-adrenal HPA axis
IL-6: Interleukin 6
